# Differences between Men and Women Enrolling in Smoking Cessation Programs Using Yoga as a Complementary Therapy

**DOI:** 10.4172/2157-7595.1000245

**Published:** 2016-06-15

**Authors:** Herpreet Thind, Ernestine Jennings, Joseph L Fava, Marie A Sillice, Bruce M Becker, Sheri J Hartman, Beth C Bock

**Affiliations:** 1 Department of Community Health and Sustainability, College of Health Sciences, University of Massachusetts Lowell, USA; 2 The Miriam Hospital, 164 Summit Avenue, Providence RI, 02906, USA; 3 Department of Psychiatry and Human Behavior, Brown Medical School, Brown University, The Miriam Hospital, USA; 4 Department of Emergency Medicine, Behavioral and Social Sciences, Alpert Medical School, Brown University, Rhode Island Hospital, USA; 5 Department of Family Medicine and Public Health, UC San Diego Moores Cancer Center, 3855 Health Sciences Drive, USA

**Keywords:** Yoga, Smoking cessation, Complementary therapies, Gender

## Abstract

This study compares the characteristics of men and women, respectively, participating in two randomized controlled pilot studies whose primary aims were to test the feasibility of yoga as a complementary therapy for smoking cessation. Participants were aged 18-65, generally healthy and were daily smokers. Analysis of variance (ANOVA) and chi-square tests examined gender differences in smoking rate, potential treatment mediators, and covariates (e.g., smoking history, health status, weight concerns, mood, and prior withdrawal symptoms). A total of 55 women and 38 men participated in the study. Differences between men and women at enrollment included: women reported significantly greater withdrawal (p<0.005), anxiety (p=0.032), and depression (p=0.027) symptoms than men. More women than men (91% *vs*. 66%) reported having been told by their doctor to quit smoking (p=0.003), had an existing smoking-related illness (33% *vs*. 13%; p=0.032), and reported smoking for weight control (15% *vs*. 0%; p=0.014). Results showed good feasibility for recruiting both men and women into a study using yoga as a complementary therapy for smoking cessation. Results also indicate that interventions may need to be tailored to meet different needs (e.g., addressing co-morbid depression) between men and women.

## Introduction

Cigarette smoking is the leading preventable cause of death in the US [1]. Differences between men and women have been reported for barriers to successful smoking cessation including, number and intensity of withdrawal symptoms, concerns about weight gain, and negative mood [2]. Research has suggested that yoga improves weight control and reduces perceived stress and negative affect among men and women [3-5]. Yogic practice includes exercises centered on regulation of breathing and focused attention, which may enhance smoking cessation outcomes by reducing stress and improving cognitive focus. However, national surveys examining yoga trends report that yoga practitioners are more likely to be women [6,7]. Thus, it is unclear whether a smoking cessation program that includes yoga as a complementary therapy would appeal to both men and women. This paper examines whether there are differences relevant to smoking cessation between men and women who enroll in smoking cessation programs that use yoga as a complementary therapy.

## Methods

This study compares characteristics of men and women who enrolled in two consecutive trials, one for women, and the other for men, to assess the feasibility of yoga for smoking cessation. Both studies were randomized, controlled trials offering cognitive behavioral therapy (CBT) for smoking cessation, plus either; 1) a twice-weekly yoga program, or 2) a general health and wellness program as a contact-control. Participants were recruited via radio, posted flyers and Internet. Eligibility criteria included: English-speaking, generally healthy, age 18-65, smoking>five cigarettes/day, not exercising regularly or practicing yoga. The yoga intervention consisted of twice-weekly, 60 min Hatha yoga sessions led by certified yoga instructors. The wellness program consisted of 60 min sessions on general health topics led by PhD-level health educators.

Measures included demographics, smoking rate and factors thought to be important in predicting cessation, including confidence in quitting, self-efficacy to abstain from smoking in high-risk situations, situational temptations to smoke, smoking-related weight concerns (Smoking Situations Questionnaire: SSQ), withdrawal symptoms related to previous quit attempts, nicotine dependence and stage of change (readiness) for quitting [8-13]. We also assessed depressive symptoms using the CESD and anxiety symptoms using the State-Trait Anxiety Inventory (STAI) and several questions concerning health history and interactions with health care providers concerning smoking [14-16]. Baseline characteristics were examined for gender differences using analysis of variance (ANOVA) or chi-square test as appropriate using IBM SPSS Statistics, version 20 [17]. Effect sizes for independent means (Cohen’s d) and independent proportions (Cohen’s h) were calculated for all significant (p<0.05) and significantly close (p.10) results [18].

## Results

A total of 351 individuals who called our center in response to advertisements (184 women, 167 men) and were screened for eligibility. Among men, the most common reasons for ineligibility were medical conditions (54%), too physically active (10%), smoking less than five cigarettes daily (9%), older than 65 years (6%), BMI>40 (5%), and current use of Nicotine replacement therapy (NRT) or enrolment in another cessation program (5%). Among women the most common reasons for ineligibility were medical conditions limiting exercise participation (58%), current major depression (47%), and smoking fewer than five cigarettes per day (15%). A total of 93 individuals (55 women and 38 men) attended the study orientation sessions, signed consent and were randomized into the studies.

Most participants were Caucasian (87%) with no differences between women and men in race or ethnicity. Women were significantly older than men (M=45.6, SD=8.3 *vs*. M=39.9, SD=13.7; p=0.025). No significant differences were observed between women and men on measures of education, marital status, household income or employment status (Table 1).

Men smoked significantly more than women (M=4.8, SD=4.2 *vs*. M=5.0, SD=1.4; p=0.045); however, there were no significant differences on smoking history variables. The mean age of smoking initiation was 18 years (SD=6.8) for women and 17 years (SD=6.7) for men with 3.2 (SD=3.4) lifetime average quit attempts for women and 3.6 (SD=3.3) for men. Nicotine dependence mean scores were also similar between men (M=5.0, SD=1.4) and women (4.8, SD=4.2). Thirty-one percent of women and 29% of men reported living with another smoker (p=0.84). Somewhat fewer women than men (39% *vs*. 58%) were in the Preparation stage of change for smoking cessation (p=0.072), however, there were no significant differences between men and women in confidence or self-efficacy for quitting smoking or in temptations to smoke (Table 1).

Smoking to control weight as assessed by SSQ was significantly greater among women than men (Mean=2.2 SD=1.1 *vs*. 1.8 SD=0.8; p=0.039), and significantly more women than men reported smoking to control their weight (15% *vs*. 0%; p=0.014). In addition, significantly more women than men reported that their doctor had advised them to quit (91% *vs*. 66%; p=0.003), and women were more likely than men to report having a medical condition or illness related to smoking (33% *vs*. 13%; p=0.032). Women reported experiencing more withdrawal symptoms during previous quit attempts compared to men (Mean=41.6 SD=11.5 *vs*. 34.9 SD=9.4; p=0.005), and reported significantly more depressive symptoms (Mean=9.9 SD=5.6 *vs*. 7.4 SD=4.6; p=0.027) and anxiety symptoms (Mean=43.3 SD=11.3 *vs*. 38.4 SD=9.1; p=0.032) compared to men.

## Discussion

The aims of this study were to examine the feasibility of yoga as a complementary therapy for smoking cessation as well as relevant characteristics for smoking cessation between men and women. Both women and men responded to advertisements for a smoking cessation program using yoga as a complementary therapy. National surveys examining trends in the use of alternative and complementary therapies suggest that more women than men practice yoga [7]. Therefore, the current study provides valuable information indicating that it is feasible to recruit both men and women into smoking cessation programs that use yoga as a complementary therapy. Moreover, participants in our studies were diverse, presenting a broad spectrum of education and income levels; this was surprising as wealthier and more educated individuals are more likely to seek cessation treatment and more likely to practice yoga [7,19]. There were several significant differences between men and women participating in our studies. Women reported feeling less ready to quit than men as assessed by the Stage of Change questionnaire, but women were equally as confident as men in their ability to quit successfully on this quit attempt. Women in the current sample reported more weight concerns, which is a known barrier to successful quitting [2]. Women also reported more withdrawal symptoms during previous quit attempts and more symptoms of depression and anxiety symptoms than men.

Yoga may be particularly useful in addressing women’s concerns about quitting smoking. Yoga has been shown to be useful in weight control and as a form of exercise that may help combat the decreased metabolic rate seen affer smoking cessation [20]. Although yoga has been associated with weight control, no studies have examined any effects of yoga practice on metabolic rate or as a means of promoting weight loss [6]. Exercise also has positive effects on depressed mood and stress, both of which are associated with weight gain [21]. Women’s greater experience of symptoms of withdrawal, anxiety and depression may suggest that women would benefit from pharmacological or behavioral therapy to address these symptoms during quit attempts. However, yoga practice has been shown to alleviate anxiety and depression, thus, yoga practice may be a particularly effective complementary therapy for women who are attempting to quit smoking [3,4]. However, men may also find a physical activity based program appealing. Efficacy trials should adopt the use of a gender-stratified analysis plan to help understand mechanism(s) for how yoga may differentially aid smoking cessation by gender [22].

Women in our studies also reported more smoking-related illnesses and were more likely to be advised by a doctor to quit smoking. This finding may reflect the gender-related age difference in our study participants; the women were older than men and morbidity accrues with advancing age. Women also tend to use more health care services than men, and this greater exposure to health care providers may have created more opportunity for health care providers to provide advice regarding quitting smoking. As there appears to be a difference between the health status of men and women seeking yoga as a complementary therapy for tobacco cessation, care management systems, such as those developed by Ciccone et al. [23], would help to direct patients to treatment resources appropriate to their needs.

## Limitations

Our overall sample size was moderate in size (N=93) with 38 men and 55 women recruited for their respective studies, and the specific characteristics of men and women recruited using other modalities and/or for larger trials may differ from those of our studies. However, our group sample sizes were sufficient to detect statistical significance for effect sizes that were in the medium range and higher range (Cohen’s d or h .40+), and effect sizes of that magnitude suggest the differences found were not trivial, and gender characteristics should be considered in planning future research for that might employ yoga interventions for smoking cessation.

Over half of those interested in the study were ineligible due to medical conditions. The majority of participants were Caucasian and so we were unable to examine whether important differences may exist among individuals from different racial and ethnic groups. This study concerns a sample of 55 women and 38 men, which is a relatively small sample for between group comparisons. Thus, although many of those comparisons achieved statistical significance, the robustness of these differences is not clear and will need to be established in larger scale trials.

## Conclusion

These results demonstrate clear differences between women and men in factors known to predict successful smoking cessation. Women were older, had experienced more withdrawal symptoms during previous quit attempts, had more symptoms of anxiety and depression, more smoking-related health problems and were more likely to have a partner who smoked. Prior research has indicated that yoga may address many of these issues, by providing exercise for weight control, ameliorating withdrawal symptoms, decreasing anxiety and depressive symptoms, and creating a social support system to counter women’s smoke-positive domestic situation. However, whether the outcomes of a yoga intervention will differ by gender, needs further investigation. These results demonstrate that it is feasible to recruit both men and women into a smoking cessation program that involves yoga, however, the motivators for men to initiate a quit attempt using yoga as a complementary therapy remain unclear. Future research is needed to identify factors that may motivate men to choose yoga in order to optimize recruitment and intervention strategies for smoking cessation for women and men.

## Figures and Tables

**Table 1:** Baseline characteristics by gender.

	Men	Women	p-value	Effect
				Size^a^
	(n=38)	(n=55)		
Age, mean (SD)	39.9 (13.7)	45.6 (8.3)	**0.025** *	0.54
Education, %			0.342	<0.35
Less than high school	10.5	12.7		
High school	31.6	21.8		
Some college	15.8	30.9		
College Graduate	42.1	34.5		
White, %	94.6	83.3	0.106	0.35
Household income, %	(n=32)	(n=49)	0.331	<0.35
Under $10,000	12.5	22.4		
$10,000-$29,999	25.0	16.3		
$30,000-$50,000	28.1	38.8		
Over $50,000	34.4	22.4		
Employed, %	71.1	54.5	0.108	0.35
Married status, %			0.527	<0.35
Single	34.2	24.5		
Married or living with significant other	36.8	47.2		
Divorced/Widowed/Separated	28.9	28.3		
Children under 18, Yes %	21.6	40.0	0.065	0.40
Age when started smoking, mean (SD)	17.0 (6.7)	18.3 (6.8)	0.349	<0.35
Smoking rate, mean (SD)	15.7 (6.8)	18.6 (8.4)	**0.045**	**0.40**
Times made serious attempt, mean (SD)	3.6 (3.3)	3.2 (3.4)	0.570	<0.35
Fagerstrom Nicotine Dependence, mean (SD)	4.8 (2.4)	5.0 (1.4)	0.585*	<0.35
	**Men**	**Women**	**p-value**	**Effect**
				**Size** ^a^
	**(n=38)**	**(n=55)**		
Stage of change for smoking, %			0.072	0.38
Contemplation	42.1	61.1		
Preparation	57.9	38.9		
Confidence to quit, mean (SD)	4.5 (1.3)	4.9 (1.3)	0.249	<0.35
Smoking Self-Efficacy Questionnaire, mean (SD)	2.6 (0.8)	2.5 (1.0)	0.730	<0.35
Smoking Situations Temptations, mean (SD)	30.5 (6.0)	32.2 (6.5)	0.220	<0.35
Doctor told to quit, Yes %	65.8	90.9	**0.003**	0.64
Disease caused by smoking, Yes %	13.2	32.7	**0.032**	0.48
Another smoker in the house. Yes %	28.9	30.9	0.84	<0.35
Withdrawal symptoms, mean (SD)	34.9 (9.4)	41.6 (11.5)	**0.005**	0.63
Smoke to control weight, Yes %	0	14.5	**0.014**	0.60
Being afraid of gaining weight kept from quitting in				0.74
past, mean (SD)	1.6 (1.0)	2.6 (1.6)	**< .001** *	
Smoking Situations Questionnaire: SSQ, mean (SD)	1.8 (0.8)	2.2 (1.1)	**0.039** *	0.41
CESD-10, mean, SD	7.4 (4.6)	9.9 (5.6)	**0.027**	0.48
STAIT total score, mean, SD	38.4 (9.1)	43.3 (11.3)	**0.032**	0.47

* Welch robust test was used because of homogeneity of variance violations

a Note that effect sizes are either given as Cohen’s *d* for independent means or Cohen’s *h* for independent proportions and these values have similar interpretations, with 0.20, 0.50, and 0.80 representing, respectively, small, medium and large effect sizes

**Significant *p* values are in bold**
